# Sense of Coherence and Work Stress or Well-Being in Care Professionals: A Systematic Review

**DOI:** 10.3390/healthcare10071347

**Published:** 2022-07-20

**Authors:** Pablo González-Siles, Manuel Martí-Vilar, Francisco González-Sala, César Merino-Soto, Filiberto Toledano-Toledano

**Affiliations:** 1 Departamento de Psicología Básica, Universitat de València, Avgda, Blasco Ibañez, 21, CP 46010 Valencia, Spain; psiles14@hotmail.com (P.G.-S.); manuel.marti-vilar@uv.es (M.M.-V.); 2 Departamento de Psicología Evolutiva y de la Educación, Universitat de València, Avgda, Blasco Ibañez, 21, CP 46010 Valencia, Spain; francisco.gonzalez-sala@uv.es; 3 Instituto de Investigación de Psicología, Universidad de San Martín de Porres, Av. Tomás Marsano 242, Lima 34, Peru; sikayax@yahoo.com.ar; 4 Unidad de Investigación en Medicina Basada en Evidencias, Hospital Infantil de México Federico Gómez, National Institute of Health, Dr. Márquez 162, Cuauhtémoc, Mexico City 06720, Mexico; 5 Unidad de Investigación Sociomédica, Instituto Nacional de Rehabilitación Luis Guillermo Ibarra, Calzada Mexico-Xochimilco 289, Arenal de Guadalupe, Tlalpan, Mexico City 14389, Mexico

**Keywords:** sense of coherence, stress, job well-being, care professionals

## Abstract

Job-related stress affects the physical and psychological health of professionals dedicated to care. This work is a systematic review that aims to determine the relationships between a sense of coherence (SOC) and work stress and well-being perceived by care professionals. The review was carried out following the PRISMA guidelines, and the search was carried out using the Web of Science (WoS), PubMed, and Scopus databases, obtaining a final selection of 41 articles. The results indicate that stress, depression, burnout, and posttraumatic stress disorder (PTSD) negatively correlate with SOC; in contrast, job satisfaction, well-being, and quality of life positively correlate with SOC. It is concluded that SOC could act as a mediating variable and as a predictor variable of these health problems.

## 1. Introduction

According to the World Health Organization (WHO), work-related stress negatively affects the psychological and physical health of workers and therefore the effectiveness of the entities for which they work [1].

Healthcare professionals, as well as other professionals dedicated to care, are considered to be one of the sectors most exposed to high levels of stress, both occasional and sustained [2]. Must be understood care professionals in a broad sense, from a biopsychosocial perspective, as those people who promote the health and autonomy of other people and provide a point of support through functional social interaction [3]. In Spain, 44.1% of doctors and nurses report living under stress, as reflected in data published by the European Foundation for the Development of Working Conditions [4]. Healthcare workers and those engaged in caregiving require a series of tools, competencies, skills, and attitudes to maintain a professional relationship with their patients [4].

Aaron Antonovsky, in his 1979 book Health, Stress and Coping, presented the salutogenic model, asking, “What is the origin of health?” The answer to this question was the sense of coherence (SOC), and this question and its answer became the fundamental core of the model [5].

The salutogenic model is opposed to the deficit model, and in this sense, it proposes a modification of the concept of health promotion instead of understanding health as a low-risk factor, it proposes that healthy factors be taken into account as those that actively promote health [6]. This model rejects the idea that stressors are intrinsically negative, introducing the possibility of healthy consequences of stressors depending on the person’s ability to resolve them [7]. Antonovsky [6] understood the most accurate conception of reality as a model in which each person, at any given time, is situated somewhere along the health/illness continuum and asked the following question: How can a person, regardless of where he or she is on this continuum, be helped to move toward better health?

To answer the latter question, the author developed two fundamental concepts: general resilience resources (GRRs) and SOC [7].

GRRs are biological, material, and psychosocial factors that come from one’s own self, from the sociocultural context, and from the physical and natural environment; they make it easier for the individual to perceive his or her life as coherent, structured, and comprehensible, sustaining the individual’s behavior [8]. A person can successfully cope with stressors by applying GRRs, thus preventing the tension caused by stressors from transforming into stress [5]. These GRRs are prerequisites for the development of SOC [9].

The optimal use of GRRs results in a particular way of perceiving life and an ability to successfully manage the infinite number of complex stressors that one has to cope with throughout life. This way of perceiving life that is a common factor to all individuals who optimally apply their GRRs was what Antonovsky called a SOC [7]. SOC is understood as a general orientation that involves seeing life as understandable, manageable, and meaningful and having the ability to cope with stressful situations [10].

According to Antonovsky [6], a person with a strong SOC, when faced with a stressor, will believe that he or she understands the challenge (understandability), will believe that he or she has the resources available to cope (manageability), and will desire and be motivated to cope with the stressor (meaningfulness). In addition, Antonovsky [6] proposed that the strength of one’s SOC is a significant factor in facilitating his or her movement toward health.

The aim of this review is to determine the different relationships between SOC and some of the symptoms derived from stress, such as depressive symptoms, anxiety, burnout, and posttraumatic stress, in caregiving professionals. At the same time, from a salutogenic perspective, the aim is to determine whether there are associations between SOC and the degree of job satisfaction, well-being, and quality of life.

## 2. Method

**Figure 1:**
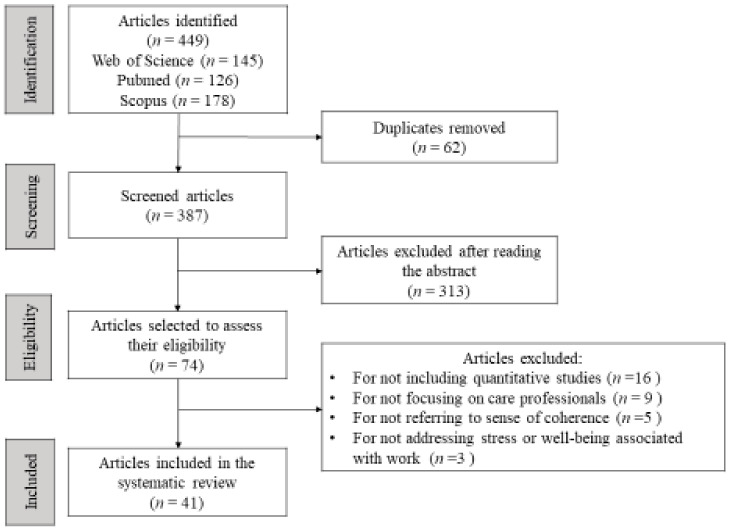
PRISMA flowchart.

This paper presents a systematic review of the published scientific literature related to SOC, both from a pathogenic perspective associated with stress and its consequences and from the perspective of the salutogenic model associated with well-being in the work of care professionals. Figure 1 shows a flow chart detailing the process carried out as proposed in the PRISMA statement [11] (Appendix A).

### 2.1. Information Sources

The systematic search was carried out between April and May 2020 in the Web of Science (WoS) and Scopus databases and in PubMed in July 2022 using the following keywords: “sense of coherence” AND “care” AND “work*”. In WoS and PubMed, the subject field, automatic search language, and time period from 1979 to 2019 were selected. In Scopus, the search was limited to the title, abstract, and keywords, as well as to articles published in the English and Spanish languages.

A total of 449 articles were obtained: 145 articles in WoS, 126 articles in PubMed, and 178 articles in Scopus. Prior to the reading of the abstracts and the selection of the articles, the inclusion and exclusion criteria were defined. After screening (see Figure 1), the final number of articles included in the review was 41.

### 2.2. Eligibility Criteria

A protocol was registered in PROSPERO and the search was conducted according to the following criteria. The identification code is CRD42022334745.

#### 2.2.1. Inclusion Criteria

The inclusion criteria were as follows: (a) articles that included research with a quantitative methodology, (b) articles that made explicit reference to the construct of SOC, (c) articles related to well-being or work-related stress in caregiving professionals, and (d) articles in English or Spanish.

#### 2.2.2. Exclusion Criteria

The exclusion criteria were (a) systematic reviews and articles that did not include quantitative research, (b) articles that did not refer to the construct of SOC, (c) articles that did not include professionals in the caregiving field, and (d) articles that did not address the topic of well-being or work-related stress.

### 2.3. Data Collection

The data to be extracted from each document were also defined in advance to ensure that the information was extracted in a uniform manner, this data was then recorded in a Microsoft Excel spreadsheet.

The spreadsheet included (1) the name of the authors, (2) title, (3) the year of publication, (4) introduction, (5) the objective, (6) the number of participants in the sample, (7) the methodology used and measures, (8) the results obtained in each study, (9) the conclusions, and (10) the limitations of each article.

### 2.4. Selection Process

The summaries of all the articles were read, 62 duplicate articles were removed, 313 articles were eliminated in an initial screening process, and only 74 articles were considered adequate for the next stage. After an analysis of the full text, 41 articles were finally selected, and 33 articles were excluded: for not including quantitative studies (*n* = 16); for not focusing on healthcare professionals (*n* = 9); for not referring to a sense of coherence (*n* = 5); for not addressing stress or well-being associated with work (*n* = 3) (Figure 1).

## 3. Results

Table 1 shows a summary of the articles selected for the review, arranged chronologically and alphabetically.

### 3.1. Pathogenic Perspective

This section includes those articles whose research focus is centered on diseases, disorders, and the variables associated with them.

#### 3.1.1. Negative Mood States

Gebrine et al. [47] explored how SOC and work values (WVs) impact midwives’ stress and perceived health, finding a significant negative correlation between SOC and work-related stress and a positive correlation between SOC and self-reported health. The results suggested that WVs could be positive mediators of the relationship between stress and health. Sarid et al. [25] investigated the effects of cognitive behavioral interventions (CBIs) on nurses’ SOC, perceived stress, and mood states. At baseline, the two groups did not differ with respect to SOC, perceived stress, and mood states; however, after the intervention in the CBI group, statistically significant changes were found in four of the psychological measures: SOC and vigor mood values increased, while perceived stress and fatigue mood values decreased significantly. In contrast, no changes were observed in the control group.

Several articles focused on studying the relationship between SOC and depression. Mackie et al. [16] examined SOC as a possible mediator of the relationships between work environment, work stress, and depression, based on the idea that participative work environments have been associated with better mental health. The results suggested that greater exposure to employee participation practices was indirectly associated with lower levels of depression through perceived job stress and SOC.

Kikuchi et al. [33] investigated the relationship between depressive status, job stress, and SOC in nurses at a Japanese general hospital, finding that SOC was inversely associated with depressive state, and age correlated positively with SOC, over-commitment and effort-esteem ratio correlated positively with depressive status, with SOC being the variable with the greatest influence on depressive status. This same inverse relationship between depression with respect to SOC, job satisfaction, and life satisfaction is pointed out by Kikuchi, Nakaya et al. [34]. Ito et al. [40] investigated whether SOC could be a predictor of future depression after years of medical residency. The results showed that the mean SOC was significantly lower in residents with recent depressive symptoms than in residents without depressive symptoms, and weekly work time was also significantly associated with new-onset depressive symptoms.

Reconciling work and family life can be a stress factor in people’s lives. In this sense, Takeuchi and Yamazaki [26] point out how high scores in SOC are related to a lower level of conflict between family life and work life, having a greater incidence on the physical and psychological health of nurses. In addition, these authors identify SOC as a damping factor for depression in family–work conflict. In this line, Makabe et al. [36] found that among the nurses who presented a greater imbalance between the duration of work and the time for private life, they presented lower job satisfaction and greater health problems. In turn, the group with a balance (50/50) between working hours and private life presents statistically significant higher scores on the SOC with respect to the rest of the groups in which there was a greater proportion of working hours 60/40, 70/30 and 80/20. 

#### 3.1.2. Burnout

Gilbar [13] noted that social workers with a strong SOC experienced less burnout due to a tendency to identify the nature of the stressor confronted and select appropriate resources for the given situation. A possible explanation for the results could be that these workers faced professional demands as challenges worthy of investment and management and felt more fulfilled. Levert et al. [15] assessed burnout and different work environment factors in a group of nurses to determine the role of SOC in the relationship between burnout and work environment. Significant positive correlations were found for emotional exhaustion and depersonalization with SOC as well as with all work environment factors; however, personal accomplishment showed very low scores and did not correlate with SOC. In the same vein, Galletta et al. [46] investigated the relationship between SOC and burnout in Italian speech-language pathologists, finding that speech-language pathologists with low SOC showed significantly higher burnout scores. Kawamura [44] found similar results with respect to a sample of attending physicians in Japan, such that lower scores on the SOC, being female, working more hours, and having fewer years of experience are related to greater burnout.

Nordang et al. [24] studied the relationship between burnout and factors such as SOC and work experience in nurses during a period of reorganizations and downsizing. The results showed a rapid and significant development of burnout in nurses with extensive experience and a significant association between burnout and low SOC scores. The most likely explanation for burnout was the stress caused by reorganizations, and low SOC scores on the first measurement may have been a risk factor. Vifladt et al. [37] studied the associations between nurses’ perception of patient safety culture, burnout, and SOC in restructured and nonrestructured intensive care units (ICUs). A positive safety culture was significantly associated with a low burnout score and strong SOC; on the other hand, restructurings were negatively associated with safety culture; however, there were no significant differences in burnout and SOC in nurses in restructured and nonrestructured ICUs. Yam and Shiu [20] in a sample of intensive care nurses point out how SOC is a protective factor in order to buffer the stress associated with the workplace as well as the level of stress in life.

Cilliers [18] includes within the salutogenic model aspects such as the sense of coherence, self-control, and resistance to stress, finding an inverse relationship between these variables and the factors of burnout—emotional exhaustion, depersonalization, and personal accomplishment, although he points out that within the salutogenic paradise, other variables must be included, such as emotional intelligence or resilience, among others. In turn, high scores on the SOC are related to greater personal fulfillment, while low scores on the SOC are related to higher levels of burnout, if the factors emotional exhaustion and depersonalization are taken into account. These same results are found by Tselabis et al. [17] in hospital nurses in Greece and by van der Colff and Rothmann [23] in a sample of nurses in South Africa. In addition, in this last study, there is a negative relationship between SOC and work stress and a positive relationship with work commitment and coping strategies.

Three articles examined the effects of personality traits and SOC on burnout in different health professions students. Skodova and Lajciakov [31] studied personality factors and the effect of psychosocial training on burnout syndrome in undergraduate health care profession students. After the completion of psychosocial training, the degree of burnout in the experimental group significantly decreased, and their SOC increased; there was no change in the control group. Skodova et al. [38] examined the effect of type D personality and other personality traits (resilience and SOC) on engagement and burnout. They found positive correlations between burnout and the negative affectivity subscale but not with the social inhibition subscale; on the other hand, SOC and resilience correlated negatively with burnout and positively with engagement.

Škodová and Bánovčinová [45] investigated the associations among type D personality components (negative affectivity and social inhibition), SOC and resilience. The results showed that the negative affectivity subscale was a significant personality predictor of resilience and SOC and that students with high levels of type D characteristics had significantly lower levels of resilience and SOC.

#### 3.1.3. Posttraumatic Stress Disorder and Secondary Traumatic Stress

Jonsson et al. [19] investigated the prevalence of PTSD among Swedish ambulance personnel and whether SOC was related to the consequences of traumatic stress. The results showed a high prevalence of PTSD symptoms and indicated that lower SOC predicted PTSD. Schäfer et al. [43] investigated the impact of SOC, resilience, and internal locus of control (LOC) in an ICU to identify factors that decreased the risk of psychopathological symptoms. Nurses showed significantly higher PTSD scores than physicians; however, there were no differences between these groups for SOC, resilience, and LOC. On the other hand, SOC was found to be the most important correlate of both general mental health problems and PTSD symptoms.

Professionals working in juvenile facilities may be affected by PTSD and SOC, as they are exposed to threatening situations at work and hear about the traumatic life events of the children in their care [39]. These professionals may suffer compassion fatigue (CF), which is composed of burnout and secondary traumatic stress (STS), or feel compassion satisfaction (CS), which refers to the feeling of professional fulfillment derived from helping others [32]. Zerach’s [32] study assessed the CF and CS of Israeli juvenile center workers (RCWs) compared to educational school workers (BSWs), as well as possible buffers related to attachment orientation, spirituality, and SOC. The research showed significant differences between RCWs and BSWs in CS but not in CF; there were also no significant differences in avoidant attachment, spirituality, or SOC. SOC and spirituality were negatively associated with STS and burnout and positively associated with CS.

Steinlin et al. [39] investigated the incidence of PTSD and PTS, as well as burnout symptoms among workers in a residential child and youth center, in addition to assessing the predictive value of SOC, self-care, and job satisfaction. Most of the workers reported having suffered a situation of threat or aggression, and half of them had symptoms of helplessness or fear after the event; with respect to STS, two-thirds reported feeling shocked after hearing traumatic experiences of the child. On the other hand, the authors found that higher SOC was associated with fewer symptoms of PTSD, STS, and burnout those work-related factors (rest, time to eat or use the bathroom, and saying “no”) were associated with fewer symptoms of burnout and PTSD; and that physical self-care factors (regular exercise, balanced nutrition, and time in nature) were associated with fewer symptoms of STS and burnout.

Two studies investigated the relationship between SOC and war trauma in Palestinian health workers. Veronese and Pepe [35] investigated whether SOC mediated the relationships between traumatic events and anxiety, social dysfunction, and loss of confidence. The results showed that SOC partially mediated the impact of trauma on both anxiety and social dysfunction with, while it fully mediated the relationship between trauma and loss of confidence, all mediation using negative correlations. Veronese and Pepe [50] asked whether intrusion and avoidance contributed to increased psychological distress, whether SOC mitigated such distress, and whether SOC equally affected different professionals. The results showed that psychological distress correlated positively with intrusion and avoidance and negatively with SOC, and the findings confirmed the mediating role of SOC in the relationship between the effects of trauma and the mental health of different professional groups.

George [12] explored the relationship between SOC strength and the perceived risks of field workers conducting home visits to home care agencies. The results showed that a strong SOC correlated with a lower frequency of encounters involving risk and with a lower perceived level of risk and that a strong SOC correlated with the ability to refuse high-risk assignments.

### 3.2. Salutogenic Perspective

This section includes those studies that place greater emphasis on variables associated with health, job satisfaction, well-being, and quality of life in general.

#### 3.2.1. Work Behavior, Job Satisfaction and Engagement

Berg and Hallberg [14] studied the effects of systematic clinical supervision on SOC, creativity, job-related stress, and job satisfaction in nurses. The intervention consisted of one year of clinical supervision combined with planned and documented individual nursing care. The results improved nurses’ perception of clinical supervision as a type of support strategy for creativity and organizational climate; however, job satisfaction and job strain did not improve with the intervention, and SOC remained stable during the intervention. On the other hand, correlation analysis suggested that a strong SOC decreased work-related strain. In this line, Ida et al. [22] find a positive relationship between SOC with job satisfaction and adaptability to the workplace. In turn, high SOC scores decrease sickness absences in nurses with more work experience. Also in Japan, Ando and Kawano [41] point out the relationship between the SOC and moral distress with job satisfaction, specifically the meaningfulness factor of the SOC and the acquiescence to patients’ “rights violations” factor in relation to moral distress are the factors that have a greater influence on the job satisfaction of psychiatric nurses.

Engström et al. [21] studied the satisfaction of the caregiving team in a nursing home before and after 6 and 12 months of the implementation of a support team using information and communication technologies (motion sensors, falls, etc.). There were improvements in psychosocial job satisfaction and quality of care in the experimental group, as well as increased factors such as internal motivation, personal development, and expectations. The results showed significant group-by-time interaction effects on factors such as family relationships, close friends, total SOC score, and the score of the SOC significance subscale.

A study by Basinska et al. [28] examined the relationship between SOC (and its components) and work-related behavior patterns. The model of work-related behaviors and experiences developed by Schaarschmidt and Fischer distinguishes four different types of work-related behavior patterns: (1) Type G, healthy; (2) type S, frugal; (3) type A, risky or overloaded; (4) type B, burnout. The results showed a relationship between a high SOC and a healthy pattern of work-related behavior (type G and type S), while a low SOC was related to type B. The authors concluded that SOC and its components appeared to be predictors of work-related behavior patterns, to a greater extent for types G and B and to a lesser extent for types S and A.

Lindmark et al. [42] explored the work environment and psychosocial health of Public Dental Service employees in a Swedish county. The results showed higher scores for SOC and job satisfaction in staff in small-sized clinics, and those younger than 40 years had higher scores for meaningfulness, happiness, job satisfaction, and autonomy. By position, clinical coordinators reported better health, more autonomy, and greater manageability. The authors concluded that since variables such as gender, age, position, or size of the workplace were influencing factors, it is important to identify the resources and processes of each workplace.

In relation to resources, Pijpker et al. [9] explored the role of conceptual, instrumental, and social learning in the relationship between SOC and key GRRs. A relationship was found between SOC and all GRRs, with work control being the most important, followed by social relationships and task importance. On the other hand, instrumental and social learning played a small mediating role between SOC and GRRs, while conceptual learning played no role. Grødal et al. [48] investigated whether a health-promoting work environment (SOC, high job resources, and low demands) strengthened affective organizational commitment (AOC) among nursing home employees. The results showed that work SOC was strongly and positively related to AOC and job resources and negatively related to job demands. Indirect effects of autonomy and supervisor support on AOC were found through job SOC; however, indirect effects with respect to the social community at work, emotional demands, and role conflict were unclear. In conclusion, the results of this study supported the hypothesis that job SOC improves AOC among nursing home employees.

#### 3.2.2. Quality of Life and General Health

Malagon-Aguilera et al. [10] examined SOC in nurses and its relationship to work engagement and overall health. Nurses with high SOC scores showed better health and greater work engagement, in addition to reporting greater social support and less work-related family conflict. The authors found that overall SOC scores and scores for understandability, manageability, and meaningfulness correlated positively with engagement and that nurses without work-related family conflict showed greater work engagement. On the other hand, Kowitlawkul et al. [49] investigated the key factors of nurses’ quality of life and work–life balance. The results showed that the key factors of high quality of life were SOC and social support; specifically, social support acted as a buffer for stress by reducing it and improving physical and psychological health and thus the quality of life. In conclusion, factors such as social support and stress management are essential to maintain nurses’ quality of life, as they play a crucial role in direct patient care.

Foureur et al. [30] tested the effectiveness of an adapted mindfulness-based stress reduction (MBSR) intervention on the psychological well-being of nurses and midwives who participated in a one-day workshop and engaged in daily meditation for 8 weeks. The quantitative results showed significant improvements in general health and SOC and lower levels of stress. Ando et al. [27] applied an intervention program based on mindfulness, based on meditation therapies, to nurses of a geriatric in Japan. The results indicate how this type of intervention increases the scores in SOC, specifically in the meaningfulness factor, improving their psychological well-being, which contributed to the increase in strategies to cope with the stress associated with work situations. Orly et al. [29] found an increase in SOC scores and a decrease in stress and fatigue in those nurses who participated in a cognitive behavioral program to reduce the level of stress associated with work compared to those nurses who did not participate in these types of interventions. The results of these studies pointed to the short-term benefits of stress for cognition, emotions, and behavior; therefore, mindfulness practice holds promise for increasing individual and occupational resilience.

## 4. Discussion

### 4.1. Pathogenic Perspective

Several investigations have found a negative correlation between SOC and stress [16,25,47], which is consistent with Antonovsky’s [6] model proposing that people with high SOC will cope better in stressful situations. Some of this research suggests that SOC may act as a mediator preventing tension from turning into stress [16], while other studies point out that WVs could be indirect positive mediators of the relationship between stress and health, without clarifying the relationship between WVs and SOC [47].

With respect to depression, studies point to a relationship between depression and low SOC scores [34,40,46]. With the diathesis-stress model of depression as a reference, SOC mediates between job strain and perceived stress, thus preventing the onset of depressive symptoms. The negative correlation between SOC and burnout also seems to be supported by different studies [13,15,17,18,20,23,24,31,32,37,38,44,46]. Schäfer et al. [43] related mental health problems in ICU workers with low SOC scores. Jonsson et al. [19], Zerach [32], Steinlin et al. [39], and Schäfer et al. [43] related psychological problems arising from traumatic events, whether in the form of PTSD or STS, with low SOC scores.

In these relationships, personality-associated aspects play an important role as relational factors or predictors of SOC, as different works have pointed out [31,38,45].

From a pathogenic perspective, the role of cognitive, affective, and instrumental strategies associated with SOC in explaining problems of anxiety, stress, depression, and burnout is key. In this sense, when the information available to the individual is perceived as disorganized, illogical, unpredictable, and uncontrollable, it hinders the person’s ability to adapt to the environment, becoming a risk factor for the appearance of problems associated with psychological health. Another important factor is the perception that people have about the availability of the necessary resources to deal with the situations they face daily in the performance of their work and the value they attach to what happens.

These three variables, comprehensibility, manageability, and meaningfulness, included in Antonovsky’s [6] model, become protective factors of psychological health and must be taken into account in the design of interventions aimed at improving the psychological health of workers.

In regard to reducing the presence of negative health symptoms, studies have pointed out that regular exercise, balanced nutrition, and time spent in nature are associated with fewer symptoms of STS and burnout [39]. At the work level, aspects such as taking a drink or bathroom break, being able to delegate responsibility, and saying “no” [39], as well as factors such as shorter workdays, rotating shifts, and regular breaks in high-risk personnel in COVID-19 [51], are associated with less burnout and PTSD symptoms.

### 4.2. Salutogenic Perspective

The studies reviewed indicate that high SOC scores are related to higher person resources, higher motivation and engagement, healthier behavior patterns, and higher job satisfaction [9,14,21,22,28,41,42,48].

GRRs are a person’s resources in his or her work environment to cope with demands and are considered precursors of SOC [9]. Several studies have related SOC to resources and work demands. Pijpker et al. [9] found an association between SOC and all GRRs, with instrumental and social learning as mediators. In the same vein, Grødal et al. [48] found that job SOC was positively related to job resources and negatively related to job demands. This study also showed that SOC was associated with AOC [48], which is consistent with the findings of Malagon-Aguilera et al. [10], who found that high SOC scores were related to higher work engagement and higher engagement, and Skodova et al. [38], who found SOC to be a predictor of engagement.

Perceiving oneself as possessing resources and having a higher commitment to the company facilitates healthier behaviors. Basinska et al. [28] found that high SOC was associated with a tendency to actively solve problems, a low tendency to give up in the face of failure, and good resilience (type G), along with an optimistic attitude toward life, high internal balance, positive attitude toward one’s professional role, and a tendency to keep distance from work-related problems (type S). In contrast, low SOC was associated with perfectionistic attitudes, low ability to distance oneself from professional tasks and to relax in free time (type A) and by a lack of inner harmony and balance, low resistance to stress (type B) [28].

Continuing along these lines, it seems logical that individuals who face problems actively and with good resilience, have an optimistic attitude, and have the ability to distance themselves adequately from problems would perceive less stress at work. This reduced stress associated with high SOC leads to higher job satisfaction [14,21] and good psychosocial health [42].

Studies have shown that high SOC scores are related to better general health and higher quality of life [10,30,49], with social support playing an important role [10,49]. In turn, interventions aimed at improving stress coping strategies in nurses from cognitive behavioral and mindfulness perspectives have proven to be beneficial [27,29], which points to the need to include this type of intervention in continuing education programs within hospitals, which may have an impact on general health and job satisfaction. In certain situations, such as in cases of pandemics, this social support may occur among healthcare professionals themselves, with trust among them playing a key role in psychological well-being [52].

Among the limitations of this study, we can mention the number of databases that have been consulted, limiting the search to only three, Web of Science, PubMed, and Scopus, as well as the time period that includes until the year 2019, just before the pandemic caused by COVID-19. Future review works would have to address the role that the sense of coherence has on health professionals during the pandemic period, between the years 2020 and 2021, which would make it possible to determine whether in exceptional situations such as those experienced in those years, the stress-absorbing and protective role with respect to psychological health that the sense of coherence has is maintained, diluted or even intensified in health personnel.

## 5. Conclusions

Based on the results of the studies analyzed, it can be concluded that strong SOC reduces stress and tension in the work environment and prevents the triggering of other mental health problems, such as depression, anxiety, burnout, PTSD, or mood, in some cases mediating personality characteristics. In addition, studies have shown that workers with high SOC scores enjoy greater job satisfaction and engagement, which extends to their lives in general, with these individuals reporting higher scores in quality of life and general health. Therefore, SOC could act as a mediating variable for general mental health problems by mobilizing coping resources and could act as a predictor variable for depression, burnout, PTSD, and even engagement and healthy behavior patterns at work.

### Practical Implications

In the current context, due to the COVID-19 pandemic, healthcare professionals are enduring extremely high amounts of stress caused by increased workload, number of hours, pressure, lack of resources, and exposure to traumatic situations. Not only has stress increased in hospital environments, all those care professionals, whether in any type of residence, juvenile center, or home care, are currently subjected to a high degree of stress. The health crisis is not yet over, and professionals continue to endure daily stress that could become chronic and lead to some of the problems discussed in this paper, such as depression, burnout, or PTSD. In this sense, as pointed out by Schmuck et al. [53], a higher SOC score in healthcare personnel during the pandemic is a protective factor in preventing mental health problems. In this situation, there is an urgent need to protect care professionals and to have reliable tools that allow preventive diagnoses to be made, and perhaps the SOC scales can be good tools for this purpose. 

## Figures and Tables

**Table 1:** Summary of the reviewed articles.

Authors/Year	Sample	Results	Limitations
George (1996) [12]	*n* = 653 workers SAD	Strong SOC correlated with fewer risk encounters, lower perceived level of risk at visits, and the ability to refuse high-risk assignments.	Of the five population strata, the sample had a weak response from strata two, three, and four. Need to revise the questionnaire items with respect to the percentage of risk involved in making home visits and levels of education.
Gilbar (1998) [13]	*n* = 102 social workers	Negative correlation between SOC and burnout.	Small sample size. Absence of comparison with social workers’ coping strategy in other fields besides health comparison.
Berg and Hallberg (1999) [14]	*n* = 22 psychiatric nurses	Improved nurse perception of supervision. Job satisfaction and stress did not improve with the intervention. Strong SOC dampened stress but not dissatisfaction with working conditions and environment.	Lack of control group. A far-reaching organizational change in the public sector during the intervention could have affected the nurses’ mood.
Levert et al. (2000) [15]	*n* = 94 psychiatric nurses	High burnout and low SOC. Low personal fulfillment. Correlation of emotional exhaustion and depersonalization with SOC and work environment.	Only factors inherent to the work environment were considered.
Mackie et al. (2001) [16]	*n* = 573 staff of a center for people with intellectual disabilities.	Greater exposure to participatory practices was indirectly associated with less depression through perceived job stress and SOC. SOC acted as a mediator preventing tension from turning into stress.	Cross-sectional study. Self-report measures were used, and little control for social desirability.
Tselebis et al. (2001) [17]	*n =* 79 nurses	There is a relationship between SOC and burnout and depression.	Not specified
Cilliers (2003) [18]	*n* = 105 general nurses	There is a negative relationship between burnout and healthy functioning. The SOC is positively related to personal fulfillment and negatively related to emotional exhaustion and depersonalization.	Not specified
Jonsson et al. (2003) [19]	*n* = 362 ambulance personnel	High prevalence of PTSD symptoms. Lower SOC predicted PTSD	There was no question about the number of traumatic events; it was assumed that workers with many years of service had a history of many stressful events.
Yam & Shiu (2003) [20]	*n* = 35 critical care nurses	Higher SOC scores are related to lower job stress and life stress.	Low internal consistency in the stress in life scale.
Engström et al. (2005) [21]	*n* = 33 residential caregivers for people with dementia	Better psychosocial satisfaction and quality of care of the experimental group.	Nonrandomized design, small samples, a dropout rate of 44% and some factors with alpha values below 0.70.
Ida et al. (2009) [22]	*n* = 614 female nurses	Higher SOC scores and a better organization of the work environment reduce sick leave	Cross-sectional study conducted in a single hospital in Japan.
Van der Colff & Rothmann (2009) [23]	*n* = 818 nurses	There is a relationship between SOC and burnout, stress, and work commitment and with coping strategies	A cross-sectional survey design, use of self-report measures and a small sample size
Nordang et al. (2010) [24]	*n* = 46 Oncology nurses	Burnout in experienced nurses. Negative correlation between SOC and burnout.	Sample size. No indication of a particularly vulnerable group of participants. Retrospective observational design.
Sarid et al. (2010) [25]	*n* = 36 Nurses	CBI group had increased SOC and vigor, decreased stress, and fatigue.	
Takeuchi & Yamazaki (2010) [26]	*n* = 138 female nurses	There is an inverse relationship between SOC and the conflict between work and family life. The SOC appears as a buffer between work and family life with respect to depression.	A cross-sectional study, self-report measures, bias in the sample as it was all women, and the use of a large number of items.
Ando et al. (2011) [27]	*n* = 28 geriatric nurses	The experimental group compared to the control group improves their scores in SOC and in psychological well-being after a mindfulness intervention based on meditation	Small sample size and no further evaluations are carried out to see if the gain scores are maintained in the experimental group compared to the control group
Basinska et al. (2011) [28]	*n* = 331 nurses	Strong correlation between SOC and a healthy pattern of behavior (type G and type S), and weak correlation of SOC with type B behavior. Type A only had the only significant correlation with SOC.	The sample included volunteers with predetermined personality traits and was restricted to nurses from large cities and large hospitals.
Orly et al. (2012) [29]	*n* = 36 nurses	There is an increase in SOC and vigor scores with respect to mood and a decrease in stress and fatigue among nurses who participate in a cognitive behavioral program.	Small sample size, other factors that are not controlled may be influencing the results.
Foureur et al. (2013) [30]	*n* = 20 nurses *n* = 20 midwives	Significant improvements in GHQ-12, SOC and DASS stress subscale after MBSR practice.	Homogeneity of the sample. Longer follow-up is required to determine the sustainability of these changes.
Skodova and Lajciakova (2013) [31]	*n* = 111 psychology and nursing students.	In the experimental group burnout decreased, SOC increased and there was no change in self-esteem.	Selection process and sample size. The validity of the observed data was limited to a set of students in selected fields. Biases associated with the researcher.
Zerach (2013) [32]	*n* = 147 RCWs *n* = 74 BSWs	There were differences between groups in compassion satisfaction but not in compassion fatigue. SOC was negatively related to burnout and secondary trauma and positively related to compassion satisfaction.	Due to the use of electronic questionnaires, the number of participants who did not agree to participate in the study could not be tracked. Use of self-report measures.
Kikuchi et al. (2014) [33]	*n* = 348 nurses	OC, overcommitment, effort-esteem ratio and age correlated with depression. SOC variable had a stronger influence and was negatively correlated with depression.	The sample size was small and included nurses from a single hospital.
Kikuchi, Nakaya et al. (2014) [34]	*n* = 347 female nurses in a general hospital	Higher depression scores are related to lower job and life satisfaction and a higher sense of coherence.	Small sample size. Other sociodemographic factors are not taken into account. There is no cut-off point to identify those subjects with a rigid SOC.
Veronese and Pepe (2014) [35]	*n* = 218 healthcare workers	SOC partially mediated the impact of trauma on both anxiety and social dysfunction, while it fully mediated the relationship between trauma and loss of confidence.	Cross-sectional design and biases in the data collection process.
Makabe et al. (2015) [36]	*n* = 1425 nurses	A greater imbalance between the working day and the time for activities in private life produces greater job dissatisfaction and a worse state of health. The group with a higher work-life balance has a higher SOC.	Cross-sectional design and non-representative sample of all hospitals in Japan.
Vifladt et al. (2016) [37]	*n* = 143 ICU nurses	Positive safety culture was associated with low burnout and strong SOC. Restructuring was negatively associated with safety culture. There were no differences in burnout and SOC according to type of ICU.	Small and unrepresentative sample size. Lack of information on employee turnover, staff/patient ratio and number of ICU beds, which were not included as covariates in the analyses.
Skodova et al. (2017) [38]	*n* = 97 nursing, midwifery, and psychology students	Positive correlation between burnout and negative affectivity subscale. SOC and resilience correlated negatively with burnout and positively with engagement.SOC was the only significant personality predictor of engagement.	Cross-sectional design, which did not allow causal interpretations of associations between variables. Age range of students.
Steinlin et al. (2017) [39]	*n* = 319 juvenile center workers	Higher SOC was associated with fewer symptoms of PTSD, STS, and burnout. Work-related self-care was associated with fewer symptoms of burnout and PTSD. Regular exercise, balanced nutrition, and time spent in nature were associated with fewer symptoms of STS and burnout.	Representativeness of the sample. The study was based on questionnaires. No personal contact with participants, symptoms could not be clinically assessed or objectified. Cross-sectional design.
Veronese and Pepe (2017) [40]	*n* = 159 healthcare workers	Psychological distress correlated positively with intrusion and avoidance, and negatively with SOC. SOC had a mediating role between the effects of trauma and mental health of different professional groups.	Small sample size. Nonrepresentativeness of participants. Lack of information on how trauma history and type of exposure may affect the efficacy of SOC as a GRR.
Ando & Kawano (2018) [41]	*n* = 130 psychiatric nurses	There is a negative relationship between moral distress with SOC and with job satisfaction.	Small and unrepresentative sample size as only one institution participated.
Ito et al. (2018) [42]	*n* = 957 resident physicians	Lower SOC in depressed residents. Weekly work time associated with depressive symptoms.	Sampling bias, those most interested in topics such as working conditions, mental health and depression responded. Relatively small number of hospitals that participated.
Lindmark et al. (2018) [43]	*n* = 486 employees dentistry	High scores in SOC and WEMS. Men had greater autonomy and manageability. Under 40, greater autonomy, happiness, job satisfaction, and meaningfulness.	Largely female and unrepresentative sample. Poor generalization of results.
Kawamura et al. (2018) [44]	*n* = 1061 attending physicians	Burnout in attending physicians is related to SOC scores after controlling for stressors and buffers	Overestimation or underestimation of the number of attending physicians with burnout. Biases in the sample. Cross-sectional design.
Pijpker et al. (2018) [9]	*n* = 481 nurses and live-in caregivers	There was a relationship between SOC and all GRRs, the most important being job control, followed by social relationships and task importance. Instrumental and social learning played a small mediating or moderating role between SOC and some GRRs.	Self-report measures, biases may have influenced the results. Cross-sectional design. The original data set was reduced to only those respondents who had fully responded to all SOC and WLPQ items.
Schäfer et al. (2018) [45]	*n* = 52 ICU and anesthesiology medical equipment	SOC, resilience, and internal LOC correlated negatively with mental health problems. SOC was the most important correlate of mental health problems and PTSD symptoms.	Small sample size. No control group. It was not possible to differentiate between respondents working in the ICU and those working in the anesthesiology unit.
Škodová and Bánovčinová (2018) [46]	*n* = 150 nursing and midwifery students.	Negative affectivity subscale was a significant personality predictor of resilience and SOC.	Cross-sectional design. Homogeneity of age and gender limited the generalizability of the results.
Galletta et al. (2019) [47]	*n* = 217 speech therapists	Negative correlation between SOC and burnout.	Participant selection bias. Cross-sectional study. Measures of information.
Gebrine et al. (2019) [48]	*n* = 228 midwives	Negative correlation of SOC and job stress and positive correlation of SOC and perceived health. Job values mediated between stress and health.	Sampling bias. Questionnaires missing data were excluded.
Grødal et al. (2019) [49]	N = 558 *n* = 166 nursing home employees	Labor SOC improved AOC among nursing home employees. The influence of specific job demands and resources was unclear.	Sample size and small group sizes were a reason for ignoring a multilevel analysis approach, which was relevant given the nested data structure.
Kowitlawkul et al. (2019) [50]	*n* = 1040 nurses	Key factors of a high quality of life were SOC and social support. Social support buffered stress by improving physical and psychological health and quality of life.	Low sample representativeness. Percentage of time spent on work and private life came from participants’ perceptions that may be underestimated or overestimated.Data were collected during an external audit.
Malagon-Aguilera et al. (2019) [10]	*n* = 156 geriatric nurses	High SOC scores associated with better health, greater work commitment, greater social support, and fewer work-related family conflicts. SOC positively correlated with dedication.	It was not possible to infer causality among SOC, well-being, and work engagement. The sample size could limit the results of the study.

## Data Availability

The raw data supporting the conclusions of this article will be made. available by the authors, without undue reservation.

## Appendix A

**Table A1:** PRISMA 2020 Checklist.

Section and Topic	Item #	Checklist Item	Location Where Item Is Reported
TITLE			
Title	1	Identify the report as a systematic review.	1
ABSTRACT			
Abstract	2	See the PRISMA 2020 for Abstracts checklist.	1
INTRODUCTION			
Rationale	3	Describe the rationale for the review in the context of existing knowledge.	1–2
Objectives	4	Provide an explicit statement of the objective(s) or question(s) the review addresses.	2
METHODS			
Eligibility criteria	5	Specify the inclusion and exclusion criteria for the review and how studies were grouped for the syntheses.	3
Information sources	6	Specify all databases, registers, websites, organisations, reference lists and other sources searched or consulted to identify studies. Specify the date when each source was last searched or consulted.	3
Search strategy	7	Present the full search strategies for all databases, registers and websites, including any filters and limits used.	3
Selection process	8	Specify the methods used to decide whether a study met the inclusion criteria of the review, including how many reviewers screened each record and each report retrieved, whether they worked independently, and if applicable, details of automation tools used in the process.	4
Data collection process	9	Specify the methods used to collect data from reports, including how many reviewers collected data from each report, whether they worked independently, any processes for obtaining or confirming data from study investigators, and if applicable, details of automation tools used in the process.	4
Data items	10a	List and define all outcomes for which data were sought. Specify whether all results that were compatible with each outcome domain in each study were sought (e.g., for all measures, time points, analyses), and if not, the methods used to decide which results to collect.	NA
	10b	List and define all other variables for which data were sought (e.g., participant and intervention characteristics, funding sources). Describe any assumptions made about any missing or unclear information.	NA
Study risk of bias assessment	11	Specify the methods used to assess risk of bias in the included studies, including details of the tool(s) used, how many reviewers assessed each study and whether they worked independently, and if applicable, details of automation tools used in the process.	NA
Effect measures	12	Specify for each outcome the effect measure(s) (e.g., risk ratio, mean difference) used in the synthesis or presentation of results.	NA
Synthesis methods	13a	Describe the processes used to decide which studies were eligible for each synthesis (e.g., tabulating the study intervention characteristics and comparing against the planned groups for each synthesis (item #5)).	4
	13b	Describe any methods required to prepare the data for presentation or synthesis, such as handling of missing summary statistics, or data conversions.	4
	13c	Describe any methods used to tabulate or visually display results of individual studies and syntheses.	4
	13d	Describe any methods used to synthesize results and provide a rationale for the choice(s). If meta-analysis was performed, describe the model(s), method(s) to identify the presence and extent of statistical heterogeneity, and software package(s) used.	NA
	13e	Describe any methods used to explore possible causes of heterogeneity among study results (e.g., subgroup analysis, meta-regression).	NA
	13f	Describe any sensitivity analyses conducted to assess robustness of the synthesized results.	NA
Reporting bias assessment	14	Describe any methods used to assess risk of bias due to missing results in a synthesis (arising from reporting biases).	-
Certainty assessment	15	Describe any methods used to assess certainty (or confidence) in the body of evidence for an outcome.	-
RESULTS			
Study selection	16a	Describe the results of the search and selection process, from the number of records identified in the search to the number of studies included in the review, ideally using a flow diagram.	3
	16b	Cite studies that might appear to meet the inclusion criteria, but which were excluded, and explain why they were excluded.	4
Study characteristics	17	Cite each included study and present its characteristics.	5–8
Risk of bias in studies	18	Present assessments of risk of bias for each included study.	-
Results of individual studies	19	For all outcomes, present, for each study: (a) summary statistics for each group (where appropriate) and (b) an effect estimate and its precision (e.g., confidence/credible interval), ideally using structured tables or plots.	NA
Results of syntheses	20a	For each synthesis, briefly summarize the characteristics and risk of bias among contributing studies.	5–8
	20b	Present results of all statistical syntheses conducted. If meta-analysis was done, present for each the summary estimate and its precision (e.g., confidence/credible interval) and measures of statistical heterogeneity. If comparing groups, describe the direction of the effect.	NA
	20c	Present results of all investigations of possible causes of heterogeneity among study results.	NA
	20d	Present results of all sensitivity analyses conducted to assess the robustness of the synthesized results.	NA
Reporting biases	21	Present assessments of risk of bias due to missing results (arising from reporting biases) for each synthesis assessed.	-
Certainty of evidence	22	Present assessments of certainty (or confidence) in the body of evidence for each outcome assessed.	-
DISCUSSION			
Discussion	23a	Provide a general interpretation of the results in the context of other evidence.	12–14
	23b	Discuss any limitations of the evidence included in the review.	NA
	23c	Discuss any limitations of the review processes used.	NA
	23d	Discuss implications of the results for practice, policy, and future research.	14
OTHER INFORMATION			
Registration and protocol	24a	Provide registration information for the review, including register name and registration number, or state that the review was not registered.	3
	24b	Indicate where the review protocol can be accessed, or state that a protocol was not prepared.	3
	24c	Describe and explain any amendments to information provided at registration or in the protocol.	-
Support	25	Describe sources of financial or non-financial support for the review, and the role of the funders or sponsors in the review.	14
Competing interests	26	Declare any competing interests of review authors.	14
Availability of data, code and other materials	27	Report which of the following are publicly available and where they can be found: template data collection forms; data extracted from included studies; data used for all analyses; analytic code; any other materials used in the review.	-

NA = not applicable.
